# Recycling of Epoxy/Fiberglass Composite Using Supercritical Ethanol with (2,3,5-Triphenyltetrazolium)_2_[CuCl_4_] Complex

**DOI:** 10.3390/polym15061559

**Published:** 2023-03-21

**Authors:** Alexander E. Protsenko, Alexandra N. Protsenko, Olga G. Shakirova, Victor V. Petrov

**Affiliations:** Department of Chemistry and Chemical Technology, Komsomolsk-na-Amure State University, 681013 Komsomolsk-on-Amur, Russia

**Keywords:** polymer composite material, fiberglass reinforced plastic, epoxy matrix, solvolysis, recycling, supercritical fluid, catalyst, fiberglass, strength

## Abstract

The widespread use of polymer composite materials (PCM) leads to an increase in non-recyclable waste. This paper discusses the feasibility of recycling fiberglass with an epoxy matrix by solvolysis in ethanol under supercritical conditions. The solvolysis process completes successfully within four hours in an environment of a pure solvent containing 10% water at a temperature of 280 °C when the solvent passes into the supercritical state. The treatment time increases up to 10 h at a process temperature of 250 °C. When using a coordination compound of copper(II) chloride with organic chloride salt having 2,3,5-triphenyltetrazolium as the counterion, having the composition of (2,3,5-triphenyltetrazolium)_2_[CuCl_4_], the treatment time is reduced. The addition of the complex of 5% by weight makes it possible to completely remove the epoxy matrix at a temperature of 250 °C for two hours. The products separated from the solvolysis liquid were studied by infrared spectroscopy. The resulting fibers were examined by thermogravimetric analysis and scanning electron microscopy. The residual strength of the recovered fibers is 98%. Thus, the resulting fibers can be reused in the composite industry. Including both for the production of decorative products and for the production of structural products made of polymer composite materials.

## 1. Introduction

Polymer composite materials (PCM) are widely used in different areas of human life in the modern world. The automobile [1,2,3], marine [4,5,6], wind power [7,8], aviation [9,10] and space industries consume the most of these materials. The life cycle of PCM parts can achieved up to 25–50 years [11,12]. Both thermoplastic polymers and thermosetting plastics can be used as a PCM matrix. Many industrial polymers used as structural materials are resistant to external environmental influences and can be preserved in nature for a long time. That is why the successful methods of recycling of these materials are necessary. One of the main problems with PCM using is that waste can be difficult to recycle. The huge amount of PCM is produced on the basis of thermosetting matrices, such as epoxy resin and polyether. Epoxy matrices were among the first to find wide application in industry. For example, all modern aviation companies, such as Airbus [13], Boeing [14], Irkut [15] and others, use epoxy binders for the production of both fiberglass and carbon fiber reinforced plastics. To reduce weight, epoxy composites are widely used in the automotive industry by companies such as BMW, Ford, Porsche, Ferrari, Toyota, Volkswagen, Mitsubishi and others [16,17]. This trend is especially intensified with the focus of the automobile industry on reducing carbon dioxide emissions. These materials are characterized by high resistance to external aggressive factors and strengthening. The most common method to recycle PCM today is shredding [18,19]. The key problem of the mechanical method is that the reinforcing filler breaks down along with the material. Additionally, the polymer matrix is not recycled in this case. Shredded plastic presents a significant environmental hazard. The second most popular method is thermal destruction. Pyrolysis is the most common method [20,21]. It is simple in terms of equipment and technology. The main disadvantage of this method is an emission of a large amount of highly volatile products in the thermal destruction of the polymer matrix. Most articles also indicate a significant decrease in the strength of the recovered reinforcing materials obtained by this method [22,23].

The chemical method is the fastest growing area of PCM processing [8,21,24,25,26,27]. In particular, the use of supercritical fluids (SCF) contributes to the destruction of the polymer matrix. The rate and depth of oxidation of substances are significantly increased when using SCF as a medium for solvolysis. This is facilitated by an increase in the kinetic energy of the molecules. At the moment, water and various organic volatile liquids have been studied as solvents. Water is a good oxidant in its supercritical state and can break down almost any polymer to carbon dioxide and water. This is due to the fact that the reactions take place under conditions of molecular dispersion of reagents located in a homogeneous high-temperature fluid of low density. The main disadvantages of this solvent are its high critical point temperature of 374 °C and pressure of 22.1 MPa and higher [26,27,28]. The undoubted disadvantage of using such supercritical fluids is the complexity of the hardware design of the units. Alcohols with a critical point of about 250 °C and 4.8 MPa and above are widely used to reduce temperatures and pressures of the process [29,30,31]. It is also known that they are used as co-solvents in a supercritical aqueous medium to reduce the temperature of the critical point of the system. The search and development of systems capable of operating efficiently at relatively low process parameters is an essential task. The use of catalysts is one of the most effective solutions to increase the efficiency of the process [8,32,33,34,35]. There is evidence of the efficient use of alkali metal compounds as catalysts for the destruction of thermosetting matrices of polymer composites. Compounds containing Na^+^ and K^+^ (especially hydroxides of these metals) are widely used in laboratory practice as catalysts for the destruction of thermosetting polymer matrices [28,36]. It was proposed to use NaOH as the catalyst in the solvolysis of epoxy in a poly(ethylene glycol) medium [36]. The way of chemical recycling of end-of-life wind turbine blades was studied [8]. The main material of the blade is a glass fiber composite, which also includes rubber, a PVC foam core and balsa wood. In this case, subcritical water as a solvent, 1-propanol as a co-solvent and KOH as a catalyst. NaOH is also used as a catalyst for the solvolysis of epoxy–vinylester matrix fiberglass reinforced in methyldiethanolamine and 3-aminopropanol [34,35]. A method is also known from [37], in which decomposition with nitric acid was employed to recover carbon fiber from carbon fiber reinforced plastic prepreg. The disadvantage of such compounds is the complexity of their removal from the reaction medium and reuse. Possible applications of organocatalysts capable of degradation are also discussed [38].

Currently, catalysts based on coordination compounds of transition 3d metals, and in particular, tetrahalide compounds of copper(II) and cobalt(II) of the composition (HL)_n_[MHal_4_] (here L is nitrogen-containing organic heterocyclic compounds, Hal is chlorine or bromine), are of great interest as new catalytic systems. Compounds of this type catalyze oxidation reactions, radical polymerization, addition of hydrogen halides to unsaturated compounds, cyclization and others [39,40,41].

In this paper, the feasibility of using an ionic-type coordination compound with tetrachlorocuprate(II) anion and organic nitrogen-containing heterocyclic cations is discussed.

## 2. Materials and Methods

### 2.1. Materials

The object of the study was a fiberglass reinforced plastic (FGRP) based on cross-woven glass fabric 1250-T30-290 (Umatex) and a two-component epoxy binder SR8100/SD8824 (Sicomin). This material and its analogues are widely used in various industries of mechanical engineering [9,15,42,43]. The nine-layer plastics were produced by vacuum assisted resin transfer molding method (VaRTM). Nine layers of woven fiberglass were laid out on a flat metal mold. The surface of the mold is treated with a semi-permanent mold release agent LOCTITE Frekote WOLO (Henkel). Auxiliary materials were also used for production: low profile resin distribution medium Greenflow 75 (Airtech), vacuum bagging sealant tape Airseal 2 (Airtech), nylon peel ply Econostitch (Airtech) and vacuum bagging film Big Blue L100 (Airtech). The impregnation of the FGRP plate was carried out from edge to edge using the SVI-20-43 (MSH Techno) installation. The resulting FGRP post was cured for 2 h at a temperature of 120 °C. 

**Figure 1:**
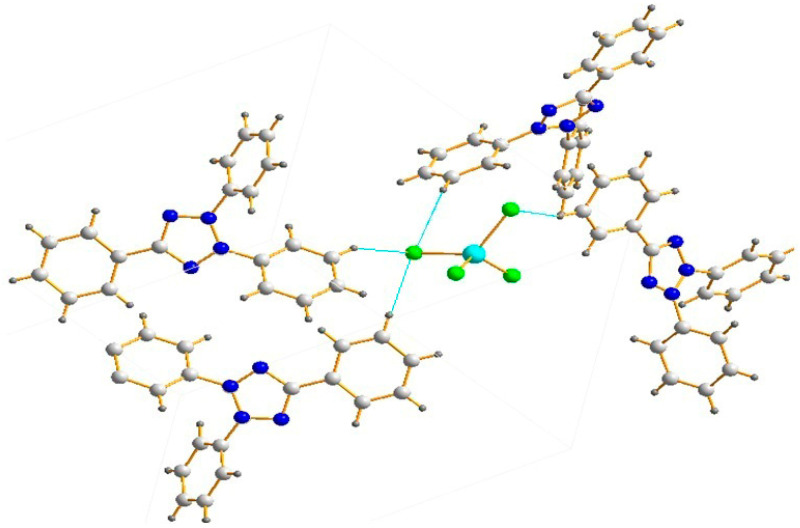
Structure of the complex (2,3,5-TPhTz)_2_[CuCl_4_] [44].

A copper(II) chloride complex with organic heterocyclic nitrogen-containing cations with the composition (2,3,5-TPhTz)_2_[CuCl_4_] (where 2,3,5-TPhTz is 2,3,5-triphenyltetrazolium) was used as a catalyst. It had been obtained previously [44] (Figure 1).

### 2.2. Methods

Solvolysis was carried out in a laboratory reactor made of fluoroplast with a volume of 25 mL. A fluoroplastic insert with a tightly sealed lid was placed into a metal flask and closed tightly. A composite sample weighting 2 g and 10 g of ethanol with a concentration of 90 wt.% was placed into the reactor. In addition, 0.2 g or 0.5 g catalyst powder (2 or 5% of the solvent weight) was also added in certain cases. Solvolysis was performed for various times in the temperature range up to 280 °C.

Thermal analysis of the samples was performed on an STA 409 PC Luxx simultaneous thermal analyzer, manufactured by NETZSCH-Gerätebau GmbH. Thermogravimetric (TG) and differential scanning calorimetric (DSC) data were recorded during the experiment. The analysis was carried out in corundum ceramic crucibles. The heating was carried out at a rate of 10 K/min in the air.

Microstructural studies were carried out using a Hitachi S-3400N scanning electron microscope (SEM) with a tungsten cathode electron gun. The measurements were carried out at an accelerating voltage of 5 kV using a secondary electron detector (SE).

The IR absorption spectra of the compounds were obtained applying an IRAffinity-1S FTIR spectrometer (Shimadzu), in the range of 400–4000 cm^−1^ with 1 cm^−1^ resolution and a 30,000:1 signal-to-noise ratio (peak-to-peak). The powder (2,3,5-TPhTz)_2_[CuCl_4_] obtained after its synthesis was used for the study. Precipitation obtained from liquids after solvolysis (during various treatment periods) after solvent removal was also studied. Samples were organized in the form of pellets in KBr.

Tensile strength of elementary glass fibers was carried out in accordance with ISO 11566:1996. Elementary fibers were isolated from the bundle of thread. The fibers were glued into a frame with a hole size of 25 × 10 mm. The optical microscope Eclipse MA200 (Nikon) was used to determine the diameter of the fiber. The breaking load was determined using the Megion 031000M test bench with a Megion 53002 dynamometer.

## 3. Results and Discussion

**Figure 2:**
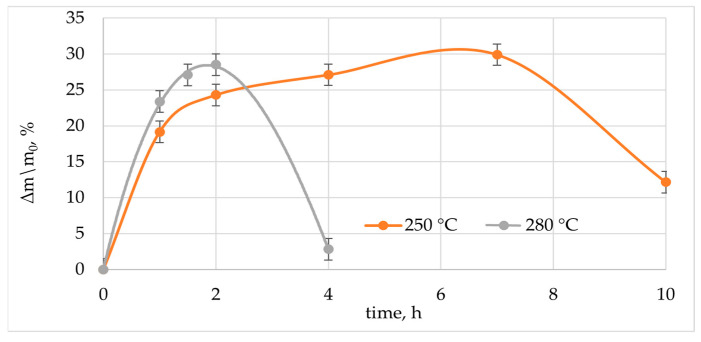
The curve of the relative mass ratio with time depending on the temperature of solvolysis.

Early studies show that alcohols under supercritical and subcritical conditions are good solvents for cured epoxy matrices [24,27,29,30,31,45,46,47]. When carrying out the experiment directly in the solvent, it was found that the destruction of the epoxy matrix was achieved at 250 °C within 10 h exposure (Figure 2). In parallel, five series of experiments were conducted in five reactors under identical conditions. The mass (m_0_, m) was determined using an analytical balance. After the selected period of time, the reactor was removed and cooled. The samples were taken out, the residual solvent was removed with filter paper, then they were weighed to determine the mass of the swollen sample (m). Measurements continued until the mass stopped changing or the sample completely delaminated. The extent of relative mass ratio for each sample was calculated as $\frac{m-{\;m}_{0}}{m_{0}}\times 100\%$.

First, the FGRP sample swelled for 7 h. The maximum swelling rate was observed in the first 2 h. The maximum speed in this section was determined as the tangent of the slope angle of the curve and was 19%/h. After this time, the swelling rate appeared to decrease. The tangent of the slope angle of the curve was 1.11%/h. It was commented in [48] that this phenomenon may reflect the difficulty with which solvent enters into the highly crosslinked polymer matrix. The maximum swelling degree of 30% was observed after 7 h of exposition in ethanol. Further exposure leads to the destruction of cross-links in the epoxy matrix, as evidenced by a decrease in the mass of the sample.

In the case of solvolysis at 280 °C in a supercritical condition, the epoxy matrix starts to absorb the solvent at a higher rate. The tangent of the angle of inclination of the curve in the area up to 1 h was 23.4%/h. After 2 h, the degree of swelling reached 28.5%. The high rate of destruction during supercritical solvolysis at 280 °C was due to the fact the solvent molecules had a higher diffusion and, consequently, a higher penetrating ability [49]. Chemical bonds between the polymer molecules were broken after filling the free volume. The recovered glass fibers were obtained after four hours.

**Figure 3:**
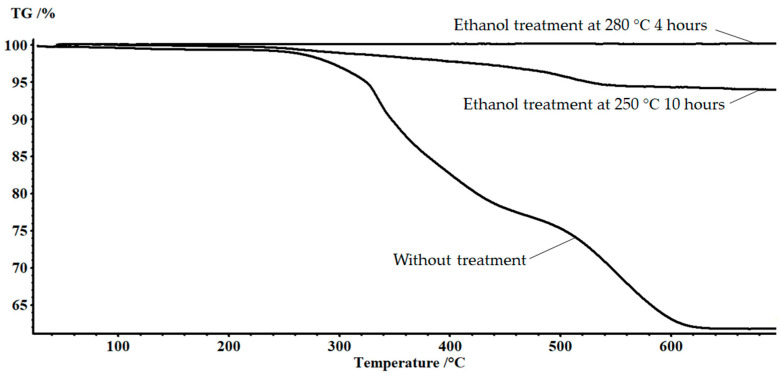
TG curves of FGRP samples.

The complete removal of the epoxy matrix was assessed in accordance with the TG data (Figure 3). The matrix content in the initial sample was 38%. The weight-loss curve consisted of three stages. The first stage corresponded to the removal of volatile low molecular weight products formed as a result of pre-curing of the sample and was 0.68%. The extrapolated beginning of the destruction process was observed at a temperature of 270 °C. A further increase in temperature contributed to the intensification of the process, which was accompanied by a change in the slope of the curve in the temperature range of 327 °C. As a result of oxidation of the polymer component of the sample, carbon remained, which burnt out in the temperature range of 500–600 °C. Based on the results of treatment in an ethanol environment at 250 °C for 10 h, the residual organic products amounted to 6%. There were three stages of weight loss on the TG curve. The first stage in the temperature range up to 200 °C was caused, apparently, by the removal of solvent residues and volatile degradation products not washed after solvolysis. This was followed by a step caused by the thermal degradation of the residues of the epoxy matrix. In the region of 500 °C, there was a change in the slope angle of the curve caused by the burnout of residual carbon. In case of solvolysis of the composite at a temperature of 280 °C for 4 h, the residual organic products were 0.1%. It became possible to obtain the recovered fiber in this way. The article [26,30] provided the details of the strength of the recovered fibers, which were identical to the primary ones. 

**Figure 4:**
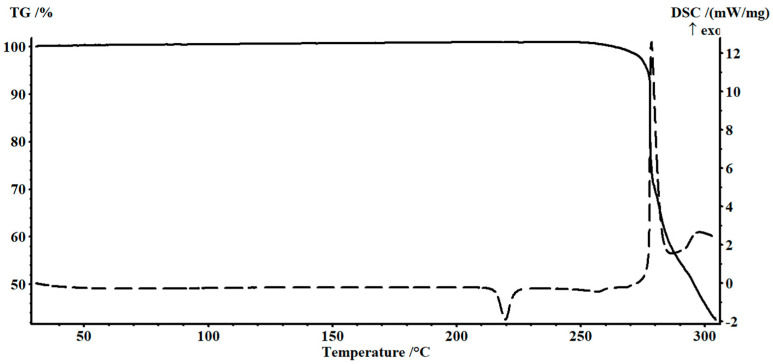
TG and DSC curves of (2,3,5-TPhTz)_2_[CuCl_4_].

In order to reduce the process temperature and increase its rate, the compound (2,3,5-TPhTz)_2_[CuCl_4_] was investigated as a catalyst. This catalyst was an anionic type complex. Based on thermal analysis data, the compound was stable at temperatures up to 277 °C (Figure 4). The selected complex had two phase transitions in the temperature range of 25–277 °C. The first one at 216 °C was monotropic (∆H_tr._ = 35.0 kJ/mol), it belonged to the “disorder/order” type and arose due to the orientational ordering of the deformed [CuCl_4_]^2−^ tetrahedron and orbital ordering in them. The transition was accompanied by thermochromism, which was a color change from yellow to orange red. The second one at 247 °C was a reversible phase transition corresponding to the melting process of the compound; ∆H_melt._ = 9.0 kJ/mol had been established. Thus, the mentioned compound could be used in the solvolysis process at 250 °C.

**Figure 5:**
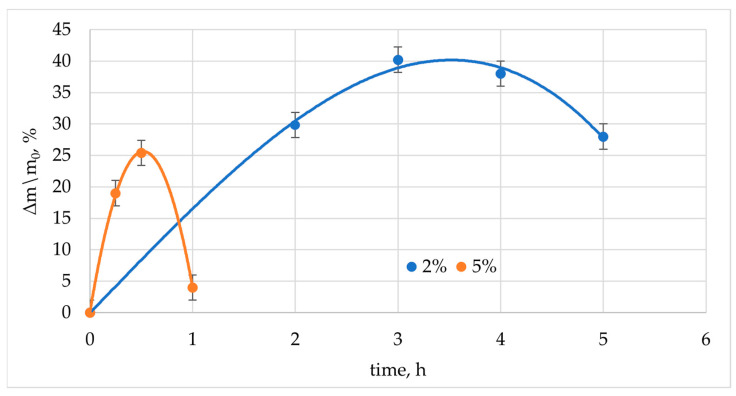
Curves of the relative mass ratio over time depending on the concentration of the complex (2,3,5-TPhTz)_2_[CuCl_4_].

**Figure 6:**
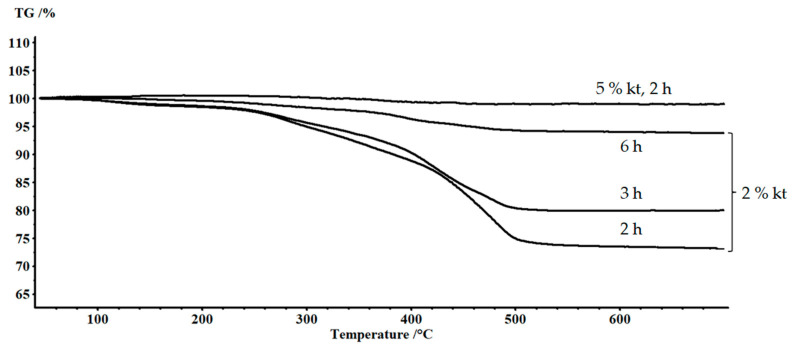
TG curves of recovered fibers depending on concentration (2,3,5-TPhTz)_2_[CuCl_4_] and treatment duration.

**Figure 7:**
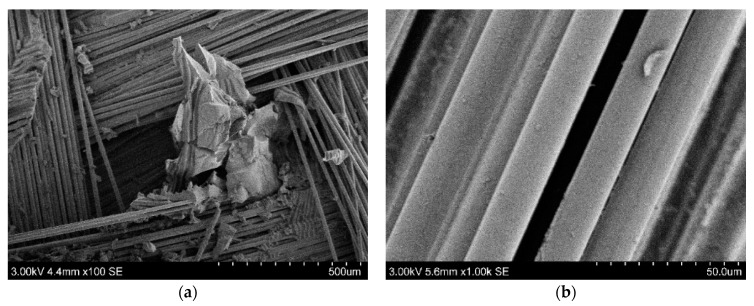

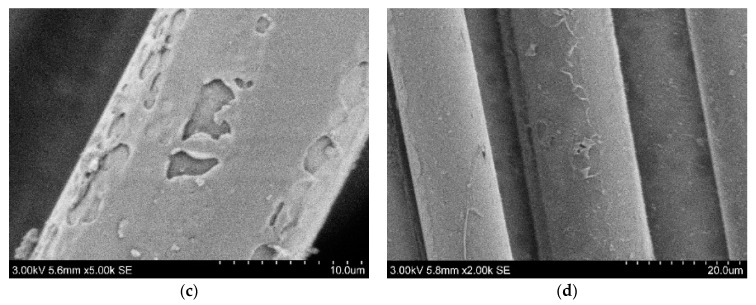
SEM microscopy of recovered fibers: (**a**) treatment with 2% catalyst for 3 h; (**b**,**c**) treatment with 2% for 6 h; (**d**) treatment with 5% for 2 h.

As a result of solvolysis in ethanol environment at 250 °C with a concentration of (2,3,5-TPhTz)_2_[CuCl_4_] 2% and 5%, it was found that the processing time was reduced. Changes in the composite weight are shown in Figure 5. The primary stage of destruction was accompanied by swelling of the epoxy matrix. After reaching the maximum swelling, a drop in the mass of the sample followed, which indicated the removal of the polymer. As a result of the experiment, recovered glass fibers were obtained. Complete removal of the epoxy matrix from the fibers was assessed using the TG method (Figure 6). With the introduction of the complex, the solvent diffusion rate increased. The maximum degree of swelling was observed after three hours of exposure to the composite in a solution containing 2% (2,3,5-TPhTz)_2_[CuCl_4_]. The maximum swelling rate observed at this site was 16.5%/h. The swelling rate in the initial period of the process was lower compared to the experiment conducted without a catalyst (Figure 2). At the same time, this rate was maintained almost over the entire swelling area of the epoxy matrix. There was no plateau preceding the swelling maximum. The phenomena presented together may indicate that the destruction of the polymer also occurred simultaneously with swelling. The maximum degree of swelling was reached after 3 h of exposure at a temperature of 250 °C and amounted to 40%. Figure 7a shows the microstructure of the solvolysis-treated PCM, consisting almost of individual fibers; however, the share of the undisturbed matrix remains high and is 20% according to TG. After six hours of treatment in this solution, the sample was completely delaminated, and, after washing with acetone, pure fibers were observed (Figure 7b). The residual content of the components of the epoxy matrix in accordance with TG was 6%. The SEM images (Figure 7c) show individual fibers in a polymer sheath up to 100 nm thick. A similar phenomenon was described earlier in [50]. In this image, there are arbitrarily shaped areas up to 5 microns at the polymer epoxy coating, in which the surface of the fiberglass is visible. 

The addition of 5% (2,3,5-TPhTz)_2_[CuCl_4_] into supercritical ethanol significantly accelerated the process. The maximum swelling (Figure 5) was achieved after 30 min. The average value of the degree of swelling at this point was 25%. The relative weight gain decreased to 4% an hour after solvolysis commencement, which indicated the destructive processes. Individual fiberglass sheets with a residual content of 1.1% decomposition products of the polymer matrix were obtained after two hours (Figure 6). The results obtained were also confirmed by SEM images. Individual fibers with insignificant inclusions of destruction products were observed. Particles ranging in size from 0.2 um to 2 um were observed (Figure 7d). Additionally, in the image, there are elongated areas formed, apparently, at the points of contact of the fibers with each other and the retention of part of the destruction products during washing.

The products of the solvolysis process obtained after the removal of ethanol from the solutions for various time periods were investigated by infrared spectroscopy. The spectrum of the complex exhibited the following vibrations of the phenyl groups: ν(C_arom_–H) at 3059 cm^−1^, ν(C=C) at 1609, 1530, 1485 cm^−1^, δ(Carom–H) at 766 и 687 cm^−1^ and tetrazole ring ν(C=N) and ν(N=N) at 1457 and 1296 cm^−1^, respectively.

A comparative analysis of infrared spectroscopy data for FGRP degradation products in supercritical ethanol in the presence of a catalyst (complex) over time (see Table 1) allowed us to conclude that the polymer dissolution process was observed during the first 30 min, while the complex composition was stable. The spectrum of the sample showed the presence of amino (~3200 cm^−1^) and hydroxy (~3400 cm^−1^) groups, as well as stretching vibrations of methyl and methylene substituents ν(C–H) at 2930 and 2860 cm^−1^. A shift of 10 cm^−1^ in the high-frequency region of the ν(C–H) band may indicate shortening of polymer chains during the reaction time. At the same time, a shift of ~30 cm^−1^ in the low-frequency region of the ν(O–H) band indicated an increased influence of hydrogen bonds on the structure of the destruction product.

With a longer exposure time, the IR spectrum of the samples showed an increase in the intensity of the bands attributed to the dissolved polymer and the destruction of the 2,3,5-triphenyltetrazolium cation, namely, the opening of the tetrazole ring, which led to a change in the spectral pattern in the area of its fingerprints. In addition, a doublet was observed in the region of 2972–3070 cm^−1^, which differed from the singlet at 3060 cm^−1^, which also indicated a change in the structure of the 2,3,5-triphenyltetrazolium cation during solvolysis for more than 60 min.

The PCM destruction process was accompanied by oxidation of organic fragments; vibrations of carbonyl groups ν(C=O) appeared in the range of 1713–1717 cm^−1^, and their intensity increased symbatically with the time of FGRP treatment.

**Figure 8:**
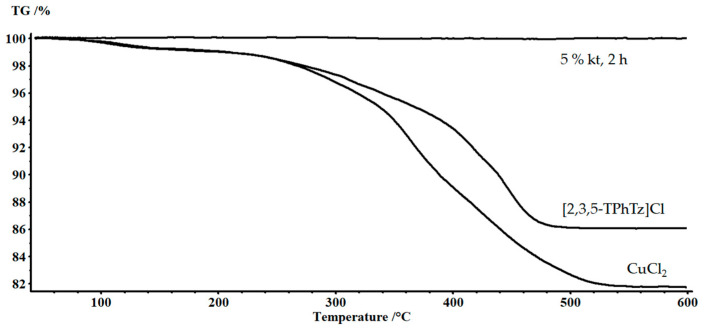
TG curves of recovered fibers obtained using various catalysts.

**Figure 9:**
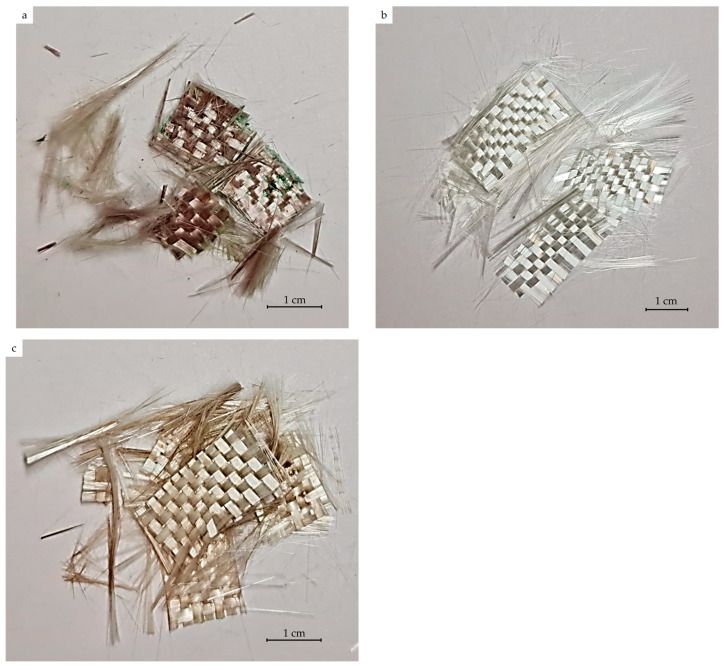
Photos of recovered fiberglass samples treated in ethanol solution with (**a**) CuCl_2_; (**b**) (2,3,5-TPhTz)_2_[CuCl_4_]; (**c**) (2,3,5-TPhTz)Cl.

To confirm the catalytic activity of the complex, rather than its individual components, experiments with CuCl_2_ and (2,3,5-TPhTz)Cl were also carried out separately. Alcohol solutions with an equimolar component content relative to 5% (2,3,5-TPhTz)_2_[CuCl_4_] solution were used. The solvolysis process took two hours. Samples of recovered fibers were studied using the TG method (Figure 8). Photos of the recovered fibers are shown in Figure 9. CuCl_2_ and (2,3,5-TPhTz)Cl separately had a negligible effect on the destruction of the epoxy matrix in a supercritical ethanol medium. The sample obtained by solvolysis in the presence of (2,3,5-TPhTz)Cl showed a residual polymer phase in the amount of 14%. The residual matrix concentration in the composite treated in the presence of CuCl_2_ was 18%. Thus, this experiment confirmed the catalytic activity of the complex compound (2,3,5-TPhTz)_2_[CuCl_4_], despite its further destruction. Similar phenomena were considered earlier [38], where the organocatalyst, N-methyl-4-piperidinol, was destroyed during SCF solvolysis.

Table 2 presents the data of the strength study of the recovered glass fibers. Thus, the residual strength of the fibers obtained using (2,3,5-TPhTz)_2_[CuCl_4_] in an SCF ethanol environment at 250 °C reached 98%. However, the strength of the fibers recovered in the SCF ethanol medium at 280 °C with twice the duration of the process reached 99%.

The difference between the strength values does not exceed 2%, which is within the instrumental error of the equipment. Thus, the introduction of a catalyst helps to efficiently reduce both the process temperature by 30 °C and the duration of solvolysis, increasing its energy efficiency. One of the key limitations of implementing the solvolysis method for large-scale use is the high cost of production and the highest energy intensity compared to other methods [51]. The use of the methodology presented in this study contributes to the promotion of this method. As a result, the duration of the processing cycle is reduced by five times. At the same time, the result is full-size woven blanks. The resulting fibers and fabrics can be used to produce structural polymer composites.

## 4. Conclusions

In this work, the ability of ethanol solution under supercritical conditions for the solvolysis of epoxy FGRP has been investigated. The recycling process worked in pure ethanol at a minimum temperature of 250 °C, and it took 10 h to be finished. At the temperature of 280 °C, the rate of the process increased. The fully recovered glass fiber was obtained after 4 h. To reduce the process temperature and increase its rate, the compound (2,3,5-TPhTz)_2_[CuCl_4_] was investigated as a catalyst. In ethanol solution, 2 or 5% catalyst powder was added. In the case of the addition of a 2% catalyst, the solvolysis of FGRP was completed in 6 h. The residual content of organic components in the fibers was 6%. In this case, the fiber was encased in a thin layer of polymer with significant traces of destruction. On the surface, there were areas of various shapes with a size of up to 5 microns of purified fiberglass. Addition of 5% compound (2,3,5-TPhTz)_2_[CuCl_4_] of the solvent weight helped to reduce the process time to 2 h at a temperature of 250 °C. On the surface of the fibers, there was an insignificant number of particles of the epoxy matrix residue after solvolysis (1.1% according to TG analysis). Thus, the recycling rate of epoxy/fiberglass composite had increased by five times. The catalytic activity of the (2,3,5-TPhTz)_2_[CuCl_4_] complex was significantly higher than that of its individual components: CuCl_2_ and (2,3,5-TPhTz)Cl. This was confirmed by the data of individual solvolysis experiments in an ethanol medium in their presence. Thus, according to TG analysis, the residual polymer phase in the presence of (2,3,5-TPhTz)Cl was 14%, and in the presence of CuCl_2_ it was 18%. The residual strength of recovered fibers was 98%. SEM and TG analyses showed the almost-cleaned surface of the fiberglass samples. FTIR spectroscopy was used to estimate the composition of the destruction liquid. However, a further investigation in this way is strongly recommended.

## Figures and Tables

**Table 1:** The main vibrational frequencies (cm^−1^) in the infrared spectra of the pure complex and PCM destruction products in supercritical ethanol in the presence of the (2,3,5-TPhTz)_2_[CuCl_4_], depending on the duration of treatment.

Complex	PCM Destruction Products Depending on the Time of Solvolysis			Assignment
(2,3,5-TPhTz)_2_[CuCl_4_] (Figure S1)	30 min Treatment (Figure S2)	60 min Treatment (Figure S3)	90 min Treatment (Figure S4)	
3446	3433	3404	3401	ν(O–H)
	3200	3200	3200	ν(N–H)
3059	3061	3059	3070 2972	ν(C_arom_–H)
	2922 2854	2927 2866	2932 2872	ν(C–H)
		1713	1717	ν(C=O)
	1632	1655	1653	δ(–NH_2_)
		1563	1559	δ(–NH–)
1609 1530 1485	1602 1528 1485	1608 1509	1604 1509	R(phenyl)
1457	1454	1455	1455	ν(C=N)
		1395	1396	ν(–N=N^−^)
1296	1297			ν(N=N)
766 721 687	767 721 686	753 716 693	753 716 690	δ(C_arom_–H)

**Table 2:** Tensile strength of the recovered fiberglass (Table S1).

Sample	T, °C	τ, h	Tensile Strength, MPa	Residual Strength, %
Initial fiberglass	-	-	2918	100
Fiberglass recovered in ethanol	280	4	2897	99
Fiberglass recovered in ethanol with (2,3,5-TPhTz)_2_[CuCl_4_] (5%)	250	2	2850	98

## Data Availability

All related additional data are available in the Supplementary Materials.

## Supplementary Materials

The following supporting information can be downloaded at: https://www.mdpi.com/article/10.3390/polym15061559/s1, Figure S1: FTIR solvolysis liquid after 30 min treatment with (2,3,5-TPhTz)_2_[CuCl_4_] 5%; Figure S2: FTIR solvolysis liquid after 60 min treatment with (2,3,5-TPhTz)_2_[CuCl_4_] 5%; Figure S3: FTIR solvolysis liquid after 90 min treatment with (2,3,5-TPhTz)_2_[CuCl_4_] 5%; Figure S4: FTIR of (2,3,5-TPhTz)_2_[CuCl_4_]; Table S1: Tensile strength of the recovered fiberglass.
