# Supplementary figures and images for: Recycling of Epoxy/Fiberglass Composite Using Supercritical Ethanol with (2,3,5-Triphenyltetrazolium)2[CuCl4] Complex
Source: Polymers (Basel). 2023 Mar 21;15(6):1559. doi: 10.3390/polym15061559 (PMC10051948; doi:10.3390/polym15061559)

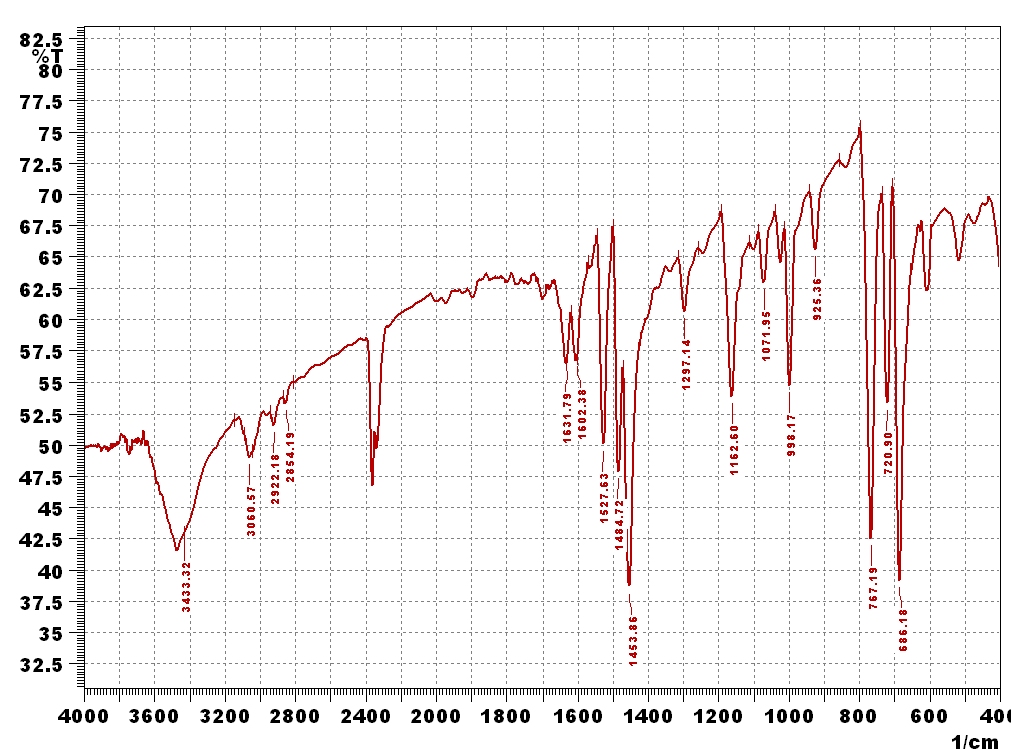

Supplement: Supplementary file 1 [file polymers-15-01559-s001.zip › Fig. S1 - FTIR solvolysis liquid after 30 min treatment with (2,3,5-TPhTz)2[CuCl4] 5%.bmp]

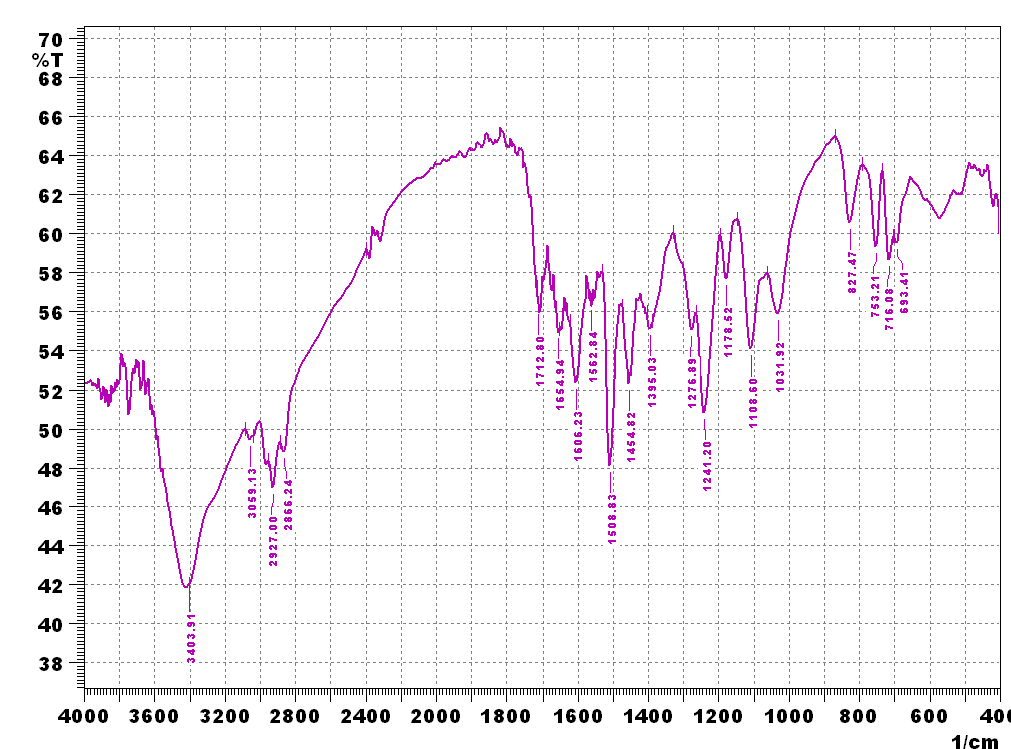

Supplement: Supplementary file 1 [file polymers-15-01559-s001.zip › Fig. S2 - FTIR solvolysis liquid after 60 min treatment with (2,3,5-TPhTz)2[CuCl4] 5%.bmp]

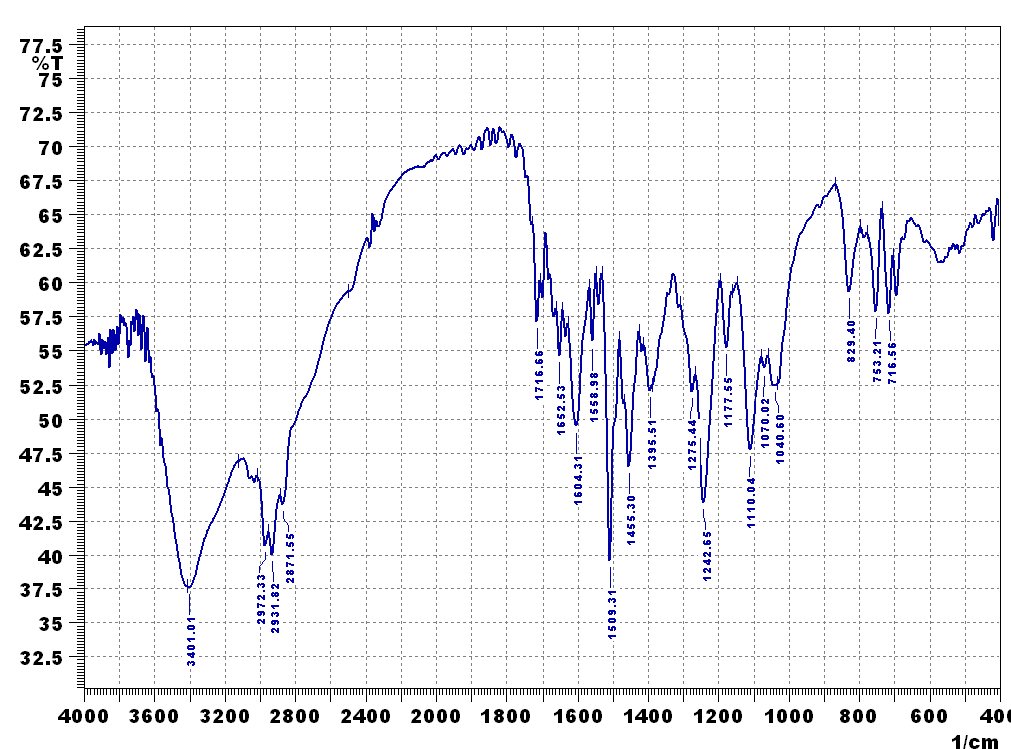

Supplement: Supplementary file 1 [file polymers-15-01559-s001.zip › Fig. S3 - FTIR solvolysis liquid after 90 min treatment with (2,3,5-TPhTz)2[CuCl4] 5%.bmp]

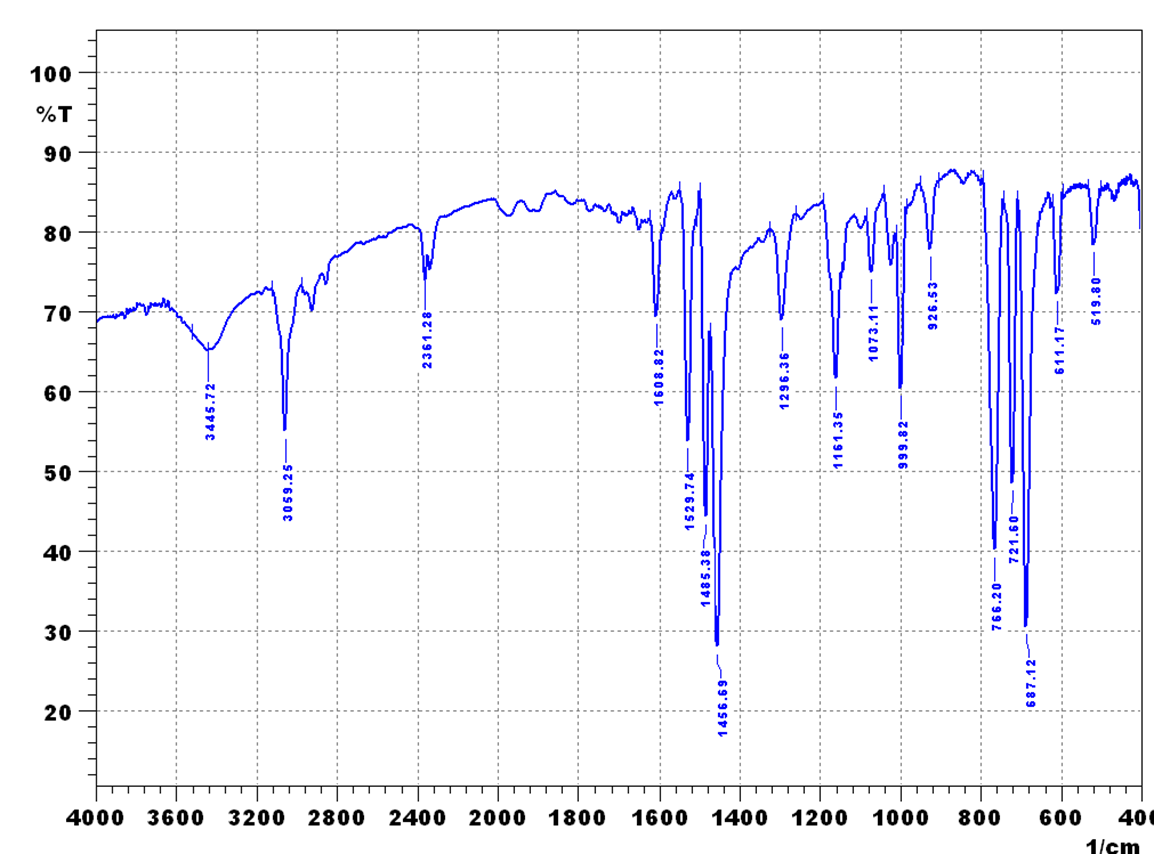

Supplement: Supplementary file 1 [file polymers-15-01559-s001.zip › Fig. S4 - FTIR of (2,3,5-TPhTz)2[CuCl4].bmp]
